# Effect of norepinephrine, vasopressin, and dopamine for survivals of the elderly with sepsis and pre-existing heart failure

**DOI:** 10.1038/s41598-024-52514-5

**Published:** 2024-01-23

**Authors:** Baohua Zhu, Jie Jiang, Hui Yu, Lan Huang, Dandan Zhou

**Affiliations:** 1 Department of Critical Care Medicine, Nanjing Central Hospital, Nanjing, Jiangsu China; 2 Department of Hematology, Affiliated Hospital of Nanjing University of Chinese Medicine, Jiangsu Province Hospital of Chinese Medicine, Nanjing, Jiangsu China; 3 Department of Pharmacy, Nanjing Central Hospital, Nanjing, Jiangsu China; 4 Department of Critical Care Medicine, Affiliated Hospital of Integrated Traditional Chinese and Western Medicine, Nanjing University of Chinese Medicine, Nanjing, Jiangsu China

**Keywords:** Cardiology, Diseases, Drug regulation

## Abstract

Our study focused on evaluating the effect of three common vasoactive drugs on the prognosis of elderly patients with sepsis and pre-existing heart failure. The Medical Information Mart for Intensive Care III database, Version 1.4, was used. Our study included critically ill older patients (aged ≥ 65 years) with sepsis and heart failure treated with vasoactive drugs. Patients were divided into norepinephrine group, norepinephrine combined with vasopressin group, and dopamine group. The baseline characteristics, primary outcome, and secondary outcome measures were compared among the three groups. In total, 1357 elderly patients were included (766 in norepinephrine group, 250 in norepinephrine combined with vasopressin group, and 341 in dopamine group). After propensity score matching, statistically significant differences in 28-d and 90-d mortality (*P* = 0.046, *P* = 0.031) were observed; meanwhile, there was a significant difference in the incidence of mechanical ventilation, AKI, and malignant arrhythmias. Cox regression analysis revealed that norepinephrine combined with vasopressin decreased 5-year survival statistically(*P* = 0.001). Multiple linear regression analysis indicated dopamine as an independent risk factor in reducing ICU and hospital length of stay (*P* = 0.001, *P* = 0.017). Logistic regression analysis showed dopamine was an independent risk factor for new-onset arrhythmias (*P* < 0.001), while norepinephrine combined with vasopressin was an independent risk factor for new-onset malignant arrhythmias (*P* < 0.001). Norepinephrine in combination with vasopressin decreased survival and increased the incidence of malignant arrhythmias in elderly sepsis patients with pre-existing heart failure. Dopamine alone reduces ICU and hospital length of stay but increases the new-onset arrhythmias.

## Introduction

Globally, life expectancy is increasing, and elderly adults comprise a growing proportion of the population^1^. In 2022, the world has 771 million people aged 65 or older, three times more than the population in 1980 (258 million). The number of elderly people is estimated to hit 994 million by 2030 and 1.6 billion by 2050. United Nations Department of Economic and Social Affairs, Population Division (2022)^2^. Sepsis is a worldwide complication of infectious processes associated with substantial morbidity and mortality. A worldwide estimate of 48.9 million cases of sepsis occurred in 2017, with 11 million deaths^3^. In mainland China, sepsis affects one in five patients admitted to intensive care units, with a 90-day mortality of 35%^4^. Based on National Health data from England, it was estimated that 77.5% of these deaths associated with sepsis occurred in individuals over the age of 75^5^. Pre-existing heart failure significantly increases mortality in patients with sepsis by about 33%.

Dopamine, norepinephrine, and vasopressin are the three most popular vasoactive drugs used clinically for the treatment of various types of shock. Current studies of vasoactive drugs are mostly focused on cardiogenic shock or septic shock alone and based on the adult population. Little attention has been paid to the protocols for utilizing vasoactive drugs in elderly sepsis patients with pre-existing heart failure. Thus, we performed a retrospective study from a large database to investigate the optimal vasoactive drug regimen in elderly septic patients with combined heart failure.

## Materials and methods

### Data source

Data for this study were obtained from the public database Medical Information Mart for Intensive Care (MIMIC III) (https://mimic.mit.edu) ^6^. The version 1.4 MIMIC-III database, maintained by the Massachusetts Institute of Technology Laboratory for Computational Physiology, contains data on patients hospitalized in an ICU at Beth Israel Deaconess Medical Center from 2001 to 2012. One of our authors, ZBH, who was responsible for data extraction, obtained free accessibility to this database after passing the examination of the National Institutes of Health (NIH) online course and gaining the certification (certification No. 36300529). Because the MIMIC-III database is a kind of publicly, available anonymized database, ethical approval was not required.

### Study population

For patients readmitted, only the first hospitalization was retained.

Inclusion criteria were as follows: (1) age of 65 years or older; (2) ICU length of stay(LOS) longer than 24 h; (3) diagnosis of sepsis and heart failure; (4) use of norepinephrine, dopamine, or norepinephrine combined with vasopressin boost as a blood pressure maintenance regimen.

Exclusion criteria included the following: (1) age less than 65 years; (2) ICU LOS less than 24 h; (3) no sepsis and no heart failure; (4) no use of norepinephrine, vasopressin, or dopamine; (5) use of a combination regimen of vasoactive drugs other than norepinephrine combined with vasopressin, such as norepinephrine combined with dopamine or vasopressin combined with dopamine.

### Data extraction and management

Heart failure was identified by International Classification of Diseases, Ninth version (ICD-9) codes: 4280, 4281, 4289, 39891, 40201, 40211, 40291, 40401, 40403, 40411, 40491, 40493, 42820, 42821, 42822, 42823, 42830, 42831, 42832, 42933, 42840, 42841, 42842, and 42843. Systolic heart failure was defined using ICD-9 codes: 42820, 42821, 42822, 42823. Diastolic heart failure was defined using ICD-9 codes:42830, 424831, 42832, 42833.

Sepsis was classified according to the criteria of the 2016 Third International Consensus Definitions for Sepsis and Septic Shock (Sepsis-3)^7^.

All-cause mortality was the primary outcome, including 7-day mortality, 28-day mortality, and 90-day mortality). Several secondary outcome indicators were included: Hospital LOS, ICU LOS, Mechanical Ventilation (MV) incidence, Acute Kidney Injury (AKI) incidence, increased heart rate, new-onset arrhythmia, new-onset malignant arrhythmia, etc. AKI was defined by the Kidney Disease: Improving Global Outcomes (KDIGO) criteria^8^. Increased heart rate was defined as the gap between the peak HR and the baseline HR extracted from ECG or ECG monitoring. The term "new-onset malignant arrhythmia" is defined as ventricular tachycardia, ventricular fibrillation, and ventricular cardiac arrest. The Vasoactive-Inotropic Score (VIS), a weighted sum of various vasopressors and inotropes, is known to be an independent predictor of adverse outcomes including ventilator days, intensive care unit length of stay, and mortality^9^.

The VIS score is therefore calculated as dopamine dose (μg kg^−1^ min^−1^) + dobutamine dose (μg kg^−1^ min^−1^) + 100 × epinephrine dose (μg kg^−1^ min^−1^) + 100 × norepinephrine dose (μg kg^−1^ min^−1^) + 10,000 × vasopressin dose (U kg^−1^ min^−1^) + 10 × milrinone dose (μg kg^−1^ min^−1^).

Baseline data were obtained based on the first data within 24 h of ICU admission. If missing, we use the last data before admission to ICU instead.

All scripts used for demographic characterization, clinical scores, and comorbidity were obtained from the GitHub website (https://github.com/MIT-LCP/mimic-code). Data extraction was performed with PostgreSQL tools (v10.0; PostgreSQL Global Development Group) using SQL.

### Statistical analysis

As a general rule, continuous variables are reported as medians and interquartile ranges, whereas categorical variables are reported as percentages. Non-parametric data were examined using the Wilcoxon rank-sum test or the Kruskal–Wallis test, whilst parametric data were studied using either analysis of variance (ANOVA). Kolmogorov–Smirnov test was used for Normality Test.

To reduce selection bias and potential confounders, propensity score matching(PSM) method^10^ was applied. Variables included in the matching include age, gender, weight, heart rate, CHF, Systolic HF, Diastolic HF, Acute Physiology Score III (APS III), Simplified Acute Physiology Score II (SAPS II), Sequential Organ Failure Assessment (SOFA), Glasgow (GCS) score, blood lactate, bilirubin, oxygenation index, platelets, blood creatinine, blood urea nitrogen, hemoglobin, etc. We set MT to 0.02, selected sampling without replacement, and used Maximize execution performance to perform PSM. Based on log-rank tests, Kaplan–Meier survival curves were analyzed. Cox regression analyses were used to analyze the significance of various variables on survival.

Multiple logistic regression modelling will be used for categorical outcomes and multiple linear regression modelling will be used for continuous variables. Variables with *P* < 0.1 at univariable analysis were included in a multivariable logistic regression model with a stepwise selection method. Statistical analyses were performed with SPSS version.25 and R version 3.5.3. Bilateral *P*-value < 0.05 was considered statistically significant.

## Results

**Figure 1:**
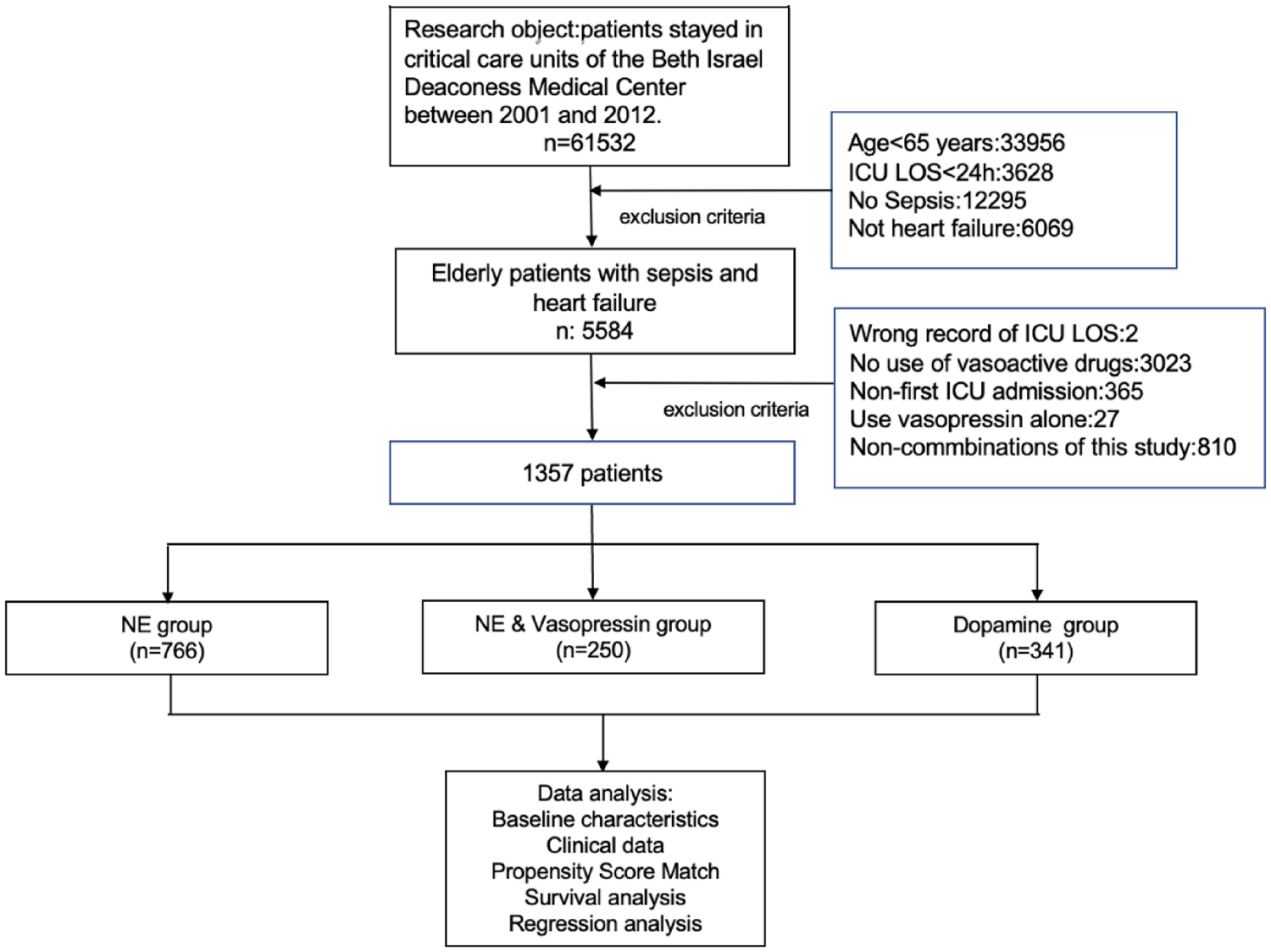
Workflow of the Study. NE-norepinephrine, LOS-length of stay.

We found 61,532 records in MIMIC-III v1.4 and finally, 1357 individuals were enrolled. 60,175 records were excluded (33,956 for patients < 65 years of Age; 3628 for ICU LOS < 24 h; 12,295 for no sepsis; 6069 for no HF; 2 for the wrong record of LOS; 3023 due to no-use of vasoactive drugs; 365 for non-first ICU admission records; 27 for vasopressin alone and 810 for non-combinations of this study) (Fig. 1).

### Baseline demographic information and clinical outcomes

Overall, 1357 patients had an average age of 79.04 years (SD, 7.46 years), 50.7% were male and 72.7% were white. Patients were divided into norepinephrine group (NE group), norepinephrine combined with vasopressin group (NE + VAS group), and dopamine group according to the vasoactive drug used. We found differences existed among the three groups in terms of demographic characteristics, comorbid diseases, and laboratory tests at ICU admission. On clinical scores that reflected the severity of the disease, there were significant differences except for the oxygenation index (PaO_2_/FiO_2_) (Table 1). To reflect some level of volemic status, the CVP values of each group of patients were shown in Table 1. Among pediatric and adult patients, VIS is known to be an independent predictor of adverse outcomes including ventilator days, intensive care unit length of stay, and mortality^11,12^, so we also analyzed VIS within 6 h, 24 h, 24 to 48 h, and 48 to 72 h after ICU admission. The primary and secondary outcomes were presented in Table 2.

**Table 1:** Baseline clinical characteristics of the study population.

Variable	NE group	NE + VAS group	Dopamine group	*P*
	(n = 766)	(n = 250)	(n = 341)	
Age(year)	79.58 (73.66, 85.08)	77.09 (71.20, 82.58)	79.68 (73.49, 85.91)	< 0.001
Gender (%male)	386 (50.4%)	142 (56.8%)	160 (46.9%)	0.058
Weight (kg)	81.75 ± 25.12	86.3 ± 29.27	81.18 ± 25.82	0.011**
Race (%white)	583 (76.1%)	191 (76.4%)	241 (70.7%)	0.128
Heart rate (bpm)	86.35 ± 18.58	90.88 ± 20.81	80.94 ± 18.48	< 0.001
*Comorbidities* (%)				
Congestive HF	611 (79.8%)	188 (75.2%)	156 (45.7%)	< 0.001
Pre-existing HF	747 (97.5%)	245 (98%)	337 (98.8%)	0.368
Systolic HF	180 (23.5%)	61 (24.4%)	52 (15.2%)	0.004
Diastolic HF	202 (26.4%)	68 (27.3%)	66 (19.4%)	0.027
Valvular disease	136 (17.8%)	54 (21.6%)	44 (12.9%)	0.019
Peripheral vascular diseases	116 (15.1%)	39 (15.6%)	49 (14.4%)	0.91
Hypertension	179 (23.4%)	62 (24.8%)	53 (15.5%)	0.006
COPD	214 (27.9%)	76 (30.4%)	96 (28.2%)	0.748
Neurological disorders	107 (14%)	23 (9.2%)	33 (9.7%)	0.041
Diabetes	275 (35.9%)	100 (40%)	109 (32%)	0.129
Hypothyroidism	126 (16.4%)	31 (12.4%)	46 (13.5%)	0.202
Chronic kidney disease	229 (29.9%)	83 (33.2%)	77 (22.6%)	0.01
Liver disease	35 (4.6%)	18 (7.2%)	11 (3.2%)	0.076
Lymphoma	21 (2.7%)	5 (2%)	4 (1.2%)	0.253
Tumor	26 (3.4%)	11 (4.4%)	9 (2.6%)	0.505
Rheumatoid arthritis	24 (3.1%)	10 (4%)	10 (2.9%)	0.744
Coagulation dysfunction	162 (21.1%)	85 (34%)	42 (12.3%)	< 0.001
*Laboratory results*				
Lactate (mmol/L)	1.7 (1.2, 2.6)	2.3 (1.5, 3.8)	1.69 (1.2, 2.7)	< 0.001
Bilirubin (mg/dL)	0.6 (0.4, 1.2)	0.8 (0.48, 1.7)	0.6 (0.3, 1.0)	< 0.001
Creatinine (mg/dL)	1.3 (0.9, 2.1)	1.5 (1.0, 2.3)	1.5 (1.0, 2.5)	< 0.001
BUN (mg/dL)	32 (21, 49)	36 (23, 51)	35 (23, 55)	0.004
PLT (10^3^/uL)	201 (142, 282)	198 (133.5, 296)	212 (162.5, 283)	0.389
Hemoglobin (g/dL)	10.12 ± 1.76	10.19 ± 1.70	10.44 ± 1.83	0.017
*Clinical scores*				
SOFA score	7 (5, 9)	9 (6, 11)	6 (4, 8)	< 0.001
APS III	55 (43, 67)	67 (52, 88)	53 (42, 65)	< 0.001
SAPS II	48 (40, 55)	55 (45, 67)	45 (37.5, 54)	< 0.001
GCS	11 (8, 14)	10 (4, 14)	13 (9, 15)	< 0.001
PaO_2_/FiO_2_	221 (146, 335)	200 (122, 306)	206 (139, 296)	0.059
Septic shock (%)	310 (40.5%)	152 (60.8%)	138 (40.5%)	< 0.001
CVP (mmHg)	11.85 ± 5.79 (n:312)	14.61 ± 6.94 (n:112)	12.44 ± 6.27 (n:124)	
CO (L/min)	4.38 ± 1.52 (n:43)	6.45 ± 9.02 (n:35)	6.05 ± 1.20 (n:2)	0.329

Data presented as n (%), mean ± SD, or median and interquartile range (IQR).

NE, norepinephrine; VAS, vasopressin; HF, heart failure; COPD, chronic obstructive pulmonary disease; BUN, blood urea nitrogen; PLT, Platelet; SOFA, sequential organ failure assessment; APS III, Acute Physiology Score III; GCS, Glasgow coma scale; SAPS II, Simplified Acute Physiology Score II, CVP, central venous pressure; CO, cardiac output.

**Using Welch’s test.

**Table 2:** Outcomes of the study population.

Variable	NE group	NE + VAS group	Dopamine group	*P*
	(n = 766)	(n = 250)	(n = 341)	
*Mortality*(%)				
7-day	88 (11.5%)	64 (25.6%)	40 (11.7%)	< 0.001
28-day	239 (31.2%)	135 (54%)	96 (28.2%)	< 0.001
90-day	341 (44.5%)	166 (66.4%)	145 (42.5%)	< 0.001
Ln (ICU-LOS(h))	5.01 ± 0.87	5.38 ± 0.94	4.76 ± 0.83	< 0.001
Ln (Hos-LOS(d))	2.38 ± 0.77	2.39 ± 0.98	2.24 ± 0.76	0.015
Mechanical Ventilation (%)	530 (69.2%)	223 (89.2%)	199 (58.4%)	< 0.001
AKI (%)	173 (22.6%)	105 (42%)	69 (20.2%)	< 0.001
New-onset arrhythmia (%)	280 (36.6%)	108 (43.2%)	172 (50.4%)	< 0.001
New-onset malignant arrhythmia (%)	30 (3.9%)	29 (11.6%)	16 (4.7%)	< 0.001
VIS6h	4.00 (0, 16.99)	5.66 (0, 30.38)	5 (0, 8.22)	0.003
VIS24h	10.02 (1, 24.02)	28.07 (3, 67.83)	5.5 (3, 10)	< 0.001
VIS48h	1.89 (0, 12)	23.92 (5.95, 67.93)	1.01 (0, 5)	< 0.001
VIS72h	0 (0, 15.10)	34.88 (6.69, 122.26)	0 (0, 4.01)	< 0.001
Fentanyl	292 (38.1%)	102 (40.8%)	72 (21.1%)	< 0.001
Propofol	287 (37.5%)	101 (40.4%)	96 (28.2%)	0.003
Midazolam	249 (32.5%)	140 (56%)	71 (20.8%)	< 0.001
Dexmedetomidine	10 (1.3%)	4 (1.6%)	1 (0.3%)	0.235
*Fluid-balance* (ml)				
24 h	395 (−541, 1862)	1640 (0, 4056)	−46 (−1173, 546)	< 0.001
48 h	0 (−1660, 1735)	1489 (−631, 4176)	−313 (−2417, 357)	< 0.001
72 h	−438 (−2944, 1151)	969 (−1568, 3598)	−1026 (−3592, 3)	< 0.001

NE, norepinephrine; VAS, vasopressin; LOS: length of stay; Hos: hospital; AKI: acute kidney injury.

VIS: Vasoactive-Inotropic Score.

### Clinical outcomes after PSM

To balance the baseline factors, we performed PSM. After 1:1 PSM, all of the groups were comparable concerning characteristics (Table 3), however, we found significant differences between 3 groups for outcome variables, especially mortality at 28 days and 90 days (Table 4).

**Table 3:** Baseline clinical characteristics after propensity score matching.

Variables	NE group	NE + VAS group	Dopamine group	*P*
	(n = 136)	(n = 136)	(n = 136)	
*Characteristics*				
Age(year)	77.33 (72.68, 82.83)	78.05 (70.96, 83.52)	77.94 (72.31, 82.23)	0.808
Gender (%male)	73 (53.7%)	76 (55.9%)	75 (55.1%)	0.952
Weight (kg)	82.44 ± 20.88	80.11 ± 21.8	80.58 ± 21.92	0.640
HR (bpm)	87.05 ± 17.6	86.13 ± 18.6	87.3 ± 20.4	0.870
CHF	105 (77.2%)	94 (69.1%)	92 (67.6%)	0.189
Pre-existing HF	133 (97.8%)	134 (98.5%)	134 (98.5%)	1.0^#^
Systolic HF	30 (22.1%)	35 (25.7%)	29 (16.2%)	0.152
Diastolic HF	38 (27.9%)	40 (29.4%)	28 (20.6%)	0.206
*Laboratory results*				
Lactate (mmol/L)	2.0 (1.3, 2.89)	2.0 (1.4, 2.8)	1.94 (1.3, 2.89)	0.735
Bilirubin (mg/dL)	0.7 (0.4, 1.38)	0.6 (0.4, 1.2)	0.6 (0.3, 1.3)	0.612
Creatinine (mg/dL)	1.4 (0.9, 2.4)	1.5 (1.0, 2.2)	1.55 (1.02, 2.5)	0.478
BUN (mg/dL)	34 (22, 50)	32.5 (21, 51)	38 (22, 54.8)	0.473
PLT (10^3^/uL)	202.5 (139.3, 276.5)	203 (132.5, 301)	201 (147.3, 275.3)	0.985
Hemoglobin (g/dL)	10.27 ± 1.72	10.25 ± 1.62	10.13 ± 1.94	0.778
*Clinical scores*				
SOFA	7 (6, 9)	7 (5, 9)	7 (5, 9)	0.616
APS III	58 (46, 71)	57 (47, 70)	57 (48, 71)	0.986
SAPS II	49 (41, 56)	48 (41, 57)	50 (41, 57)	0.835
GCS	12 (8, 14)	11 (8, 14)	11 (8, 14)	0.708
PaO_2_/FiO_2_	235 (150, 338)	211 (124.5, 316)	199 (135, 308)	0.702
Septic shock (%)	70 (51.5%)	72 (52.9%)	66 (48.5%)	0.760

NE, norepinephrine; VAS, vasopressin; HR: heart rate; CHF: congestive heart failure; BUN: blood urea nitrogen; PLT: platelet; SOFA, sequential organ failure assessment; APS III, Acute Physiology Score III; GCS: Glasgow Coma Scale.

SAPS II, Simplified Acute Physiology Score II.

**Table 4:** Clinical outcomes after propensity score matching.

Variables	NE group	NE + VAS group	Dopamine group	*P*
	(n = 136)	(n = 136)	(n = 136)	
*Mortality* (%)				
7-d	18 (13.2%)	25 (18.4%)	19 (14%)	0.472
28-d	51 (37.5%)	69 (50.7%)*	52 (38.2%)	0.046
90-d	65 (47.8%)	86 (63.2%)*	70 (51.5%)	0.031
Ln(ICU-LOS(h))	5.19 ± 0.91	5.44 ± 0.88	4.92 ± 0.81	< 0.001
Ln(Hos-LOS(d))	2.27 ± 0.84	2.49 ± 0.87	2.23 ± 0.75	0.017
Mechanical ventilation(%)	90 (66.2%)	115 (84.6%)*	91 (66.9%)	0.001
AKI (%)	21 (15.4%)	54 (39.7%)*	25 (18.4%)	< 0.001
New-onset arrhythmia (%)	45 (33.1%)	54 (39.7%)	65 (47.8%)	0.051
New-onset malignant arrhythmia (%)	7 (5.1%)	17 (12.5%)*	3 (2.2%)	0.002^#^
VIS6h	6.46 (0, 20.01)	0 (0, 24)	5 (0, 10)	0.045
VIS24h	15 (5, 30)	17.41 (0, 63.65)	5.89 (2.5, 10.56)	< 0.001
VIS48h	3.49 (0, 15)	17.78 (0, 49.85)	0.8 (0, 5)	< 0.001
VIS72h	0 (0, 18.02)	36.2 (8.72, 123.6)	0 (0, 5)	< 0.001
Fentanyl	48 (35.3%)	52 (38.2%)	34 (25%)	0.051
Propofol	47 (34.6%)	55 (40.4%)	40 (29.4%)	0.161
Midazolam	45 (33.1%)	72 (52.9%)	31 (22.8%)	< 0.001
Dexmedetomidine	3 (2.2%)	2 (1.5%)	1 (0.7%)	0.875^#^
*Fluid-balance* (ml)				
24 h	395 (−541, 1862)	991 (−105, 4056)	0 (−1099, 1064)	< 0.001
48 h	−31 (−1903, 1592)	552 (−958, 3587)	−163 (−2621, 1118)	< 0.001
72 h	−687 (−3391, 915)	0 (−2192, 3267)	−944 (−3702, 394)	0.001

NE, norepinephrine; VAS, vasopressin; LOS, length of stay; Hos-LOS, hospital length of stay; AKI, acute kidney injury; VIS, Vasoactive-Inotropic Score.

Significant difference between this group and the other 2 groups;# using Fish’s exact test.

### Five-year survival analysis and risk factor

**Figure 2:**
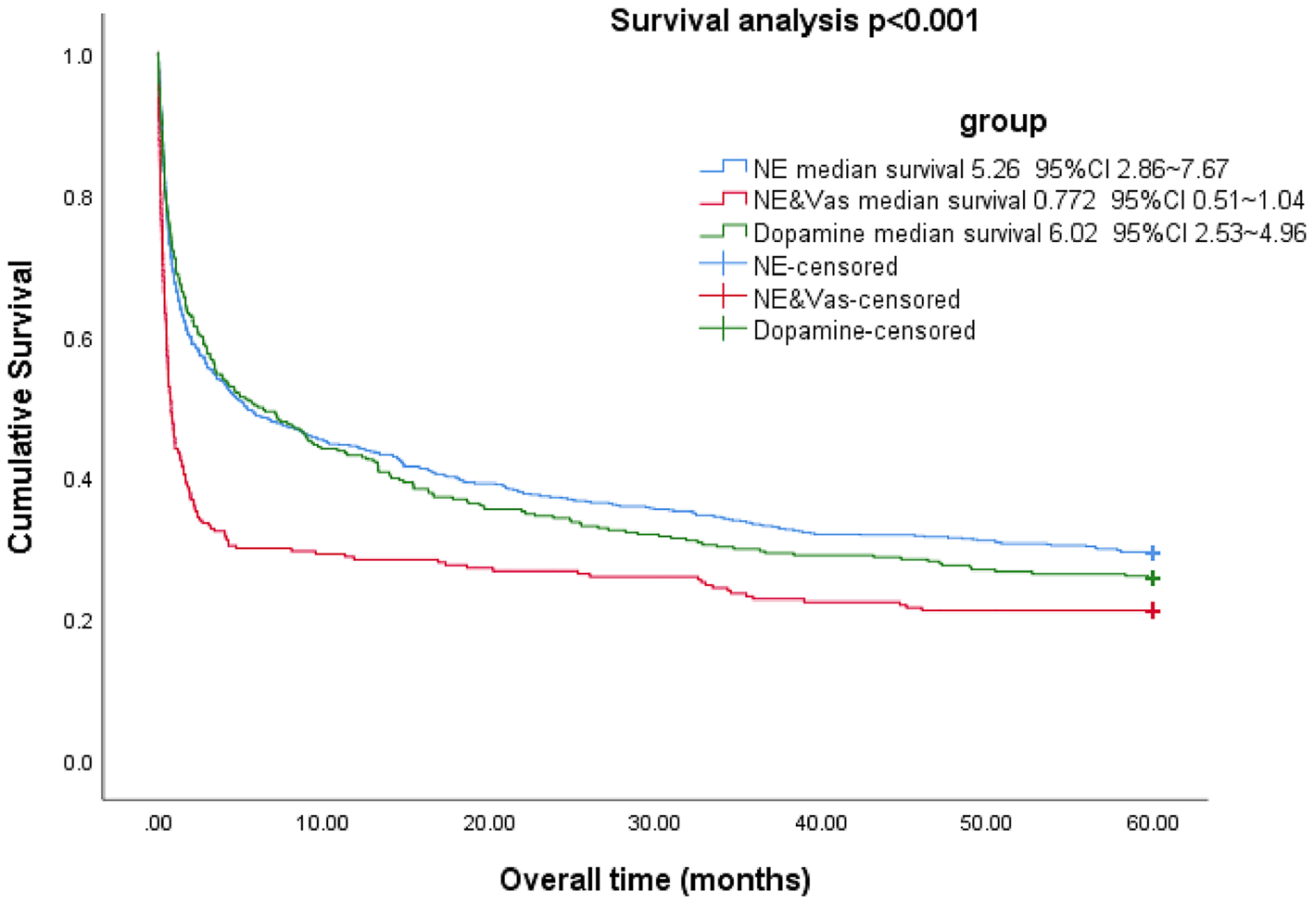
Five-year survival analysis in NE, NE + VAS and dopamine groups were 29.4%, 21.2%, 25.8%, respectively. NE-norepinephrine, VAS-vasopressin.

5-year survival analysis of all 1367 patients suggested NE group, dopamine group, and NE + VAS group had significantly different cumulative survival rates (*P* < 0.001, Fig. 2). After Cox regression models, based on our study population, the combined norepinephrine and vasopressin decreased 5-year survival (HR = 1.346, *P* = 0.001) (Table 5).

**Table 5:** Risk factors associated with 5-year mortality in the study population.

	Unadjusted (n = 1357)	*P*	Adjusted (n = 1357)	*P*
	HR (95% CI)		HR (95% CI)	
Gender (male)	0.958 (0.845–1.085)	0.496		
Age	1.016 (1.008–1.025)	< 0.001	1.016 (1.008–1.025)	< 0.001
Weight	0.996 (0.993–0.999)	0.020		0.054
Race (white)	1.041 (0.901–1.201)	0.587		
Group (NE reference)				
NE + VAS	1.524 (1.294–1.794)	< 0.001	1.381 (1.163–1.840)	< 0.001
Dopamine	1.055 (0.908–1.225)	0.484	1.146 (0.975–1.346)	0.098
SOFA	1.056 (1.035–1.078)	< 0.001		0.216
APS III	1.016 (1.013–1.019)	< 0.001	1.012 (1.009–1.015)	< 0.001
GCS	0.975 (0.960–0.990)	0.001		0.354
Lactate	1.053 (1.018–1.088)	0.003		0.579
PO_2_/FiO_2_	1.000 (0.999–1.000)	0.048		0.265
Bilirubin	1.038 (1.008–1.068)	0.012		0.193
PLT	1.000 (0.999–1.001)	0.960		
Creatinine	1.105 (1.062–1.150)	< 0.001		0.200
BUN	1.007 (1.005–1.010)	< 0.001	1.004 (1.002–1.007)	0.001
Hemoglobin	0.955 (0.921–0.990)	0.012		0.091
HR	1.007 (1.003–1.010)	< 0.001	1.005 (1.002–1.008)	0.004
CHF	1.474 (1.279–1.699)	< 0.001	1.367 (1.171–1.595)	< 0.001
Systolic HF	0.836 (0.715–0.978)	0.023	0.755 (0.640–0.890)	0.001
Diastolic HF	0.857 (0.739–0.993)	0.037	0.724 (0.619–0.846)	< 0.001

HR: Hazard ratios; CI: Confidence interval; NE, norepinephrine; VAS, vasopressin; SOFA, sequential organ failure assessment; APS III, Acute Physiology Score III; GCS: Glasgow coma scale; PLT: Platelet; BUN, blood urea nitrogen; HR: heart rate; CHF: congestive heart failure.

Univariate analyses with enter method were performed. For multivariate analysis, a forward stepwise selection method was used with covariates showing *P*-value of less than 0.10 in the univariate analyse.

### Association with ICU LOS/Hospital LOS

As secondary endpoints, we focused on ICU LOS and Hospital LOS. According to a multivariate linear retrospective model, dopamine alone may shorten ICU LOS and Hospital LOS (Tables 6, 7).

**Table 6:** Association with ICU length of stay among the three groups.

Parameter	Univariate(n = 1357)			Multivariate (n = 1357, stepwise elimination)		
	Unstandardized B	95% CI	*P*	unstandardized B	95% CI	*P*
Gender (male)	0.047	−0.048 to 0.143	0.330			
Age	−0.016	−0.023 to −0.010	< 0.001	−0.014	−0.020 to −0.008	< 0.001
Weight	0.001	−0.001 to 0.003	0.405			
Race (white)	−0.057	−0.166 to 0.053	0.313			
Group (NE reference)						
NE + VAS	0.379	0.254–0.504	< 0.001	0.296	0.172–0.420	< 0.001
Dopamine	−0.239	−0.350 to −0.127	< 0.001	−0.187	−0.296 to −0.077	0.001
SOFA	−0.003	−0.018 to 0.012	0.675			
APS III	−0.001	−0.003 to 0.002	0.623			
GCS	−0.048	−0.059 to −0.036	< 0.001	−0.038	−0.050 to −0.027	< 0.001
Lactate	0.013	−0.013 to 0.039	0.327			
PO_2_/FiO_2_	< 0.001	−0.001 to 0.001	0.202			
Bilirubin	0.002	−0.019 to 0.023	0.858			
PLT	< 0.001	−0.001 to 0.001	0.063			0.190
Creatinine	−0.038	−0.073 to −0.004	0.030	−0.040	−0.073 to −0.007	0.018
BUN	< 0.001	−0.002 to 0.002	0.885			
Hemoglobin	−0.010	−0.037 to 0.017	0.466			
HR	0.002	0.001–0.005	0.084			0.914
CHF	0.116	0.012–0.220	0.029			0.182
Pre-existing HF	−0.294	−0.629 to 0.041	0.085			0.096
Systolic HF	−0.069	−0.185 to 0.047	0.244			0.073
Diastolic HF	−0.103	−0.214 to 0.007	0.066			0.080

CI: Confidence interval; NE, norepinephrine; VAS, vasopressin; SOFA, sequential organ failure assessment; APS III, Acute Physiology Score III; GCS: Glasgow coma scale; PLT: Platelet; BUN, blood urea nitrogen; HR: heart rate; CHF: congestive heart failure.

Variables with *P* < 0.1 at univariable analysis were included in a multivariable logistic regression model with a stepwise selection method.

**Table 7:** Association with hospital length of stay among the three groups.

Parameter	Univariate(n = 1357)			Multivariate (n = 1357, stepwise elimination)		
	unstandardized B	95% CI	*P*	unstandardized B	95% CI	*P*
Gender (male)	0.039	−0.048 to 0.125	0.379			
Age	−0.018	−0.024 to −0.012	< 0.001	−0.016	−0.022 to −0.011	< 0.001
Weight	0.001	−0.001 to 0.003	0.437			
Race(white)	−0.101	−0.201 to −0.002	0.046			
Group (NE reference)						
NE + VAS	0.012	−0.104 to 0.128	0.844	0.036	−0.081 to 0.153	0.549
Dopamine	−0.145	−0.248 to −0.041	0.006	−0.131	−0.232 to −0.030	0.017
SOFA	−0.021	−0.034 to −0.007	0.003			
APS III	−0.005	−0.007 to −0.003	< 0.001	−0.007	−0.009 to −0.005	< 0.001
GCS	−0.017	−0.028 to −0.006	0.002	−0.027	−0.038 to −0.015	< 0.001
Lactate	−0.007	−0.031 to 0.016	0.534			
PO_2_/FiO_2_	0.001	−0.001 to 0.001	0.520			
Bilirubin	−0.005	−0.024 to 0.014	0.598			
PLT	0.001	−0.001 to 0.001	0.022			
Creatinine	−0.031	−0.063 to 0.001	0.049			
BUN	−0.002	−0.003 to 0.001	0.060			
Hemoglobin	−0.013	−0.038 to 0.011	0.282			
HR	−0.001	−0.003 to 0.001	0.435			
CHF	0.001	−0.095 to 0.094	0.992			
Pre-existing HF	−0.219	−0.524 to 0.085	0.158			
Systolic HF	−0.055	−0.160 to −0.050	0.308			
Diastolic HF	−0.049	−0.149 to 0.051	0.337			

CI: Confidence interval; NE, norepinephrine; VAS, vasopressin; SOFA, sequential organ failure assessment; APS III, Acute Physiology Score III; GCS: Glasgow coma scale; PLT: Platelet; BUN, blood urea nitrogen; HR: heart rate; CHF: congestive heart failure.

Variables with *P* < 0.1 at univariable analysis were included in a multivariable logistic regression model with a stepwise selection method.

Additionally, as we all know, there were more new-onset arrhythmias observed in patients treated with dopamine than norepinephrine (high-quality evidence)^13^. So we used a logistic regression model to evaluate new-onset arrhythmias incidence and new-onset malignment arrhythmias among the three groups (Tables 8, 9).

**Table 8:** Association with new-onset arrhythmias among the three groups.

Parameter	Unadjusted (n = 1357)	*P*	Adjusted (n = 1357)	*P*
	OR (95% CI)		OR (95% CI)	
Gender (male)	0.919 (0.741–1.141)	0.445		
Age	0.992(0.978–1.007)	0.308		
Weight	0.995 (0.990–1.001)	0.050		0.261
Race(white)	0.788 (0.616–1.009)	0.059		0.410
Group (NE reference)				
NE + VAS	1.320 (0.988–1.765)	0.061	1.331 (0.972–1.821)	0.074
Dopamine	1.767 (1.364–2.287)	< 0.001	1.553 (1.178–2.047)	0.002
SOFA	0.960 (0.927–1.003)	0.319		
APS III	0.997 (0.992–1.002)	0.231		
GCS	0.966 (0.940–0.992)	0.011	0.967 (0.939–0.996)	0.026
Lactate	1.087 (1.025–1.154)	0.006		0.085
PO_2_/FiO_2_	1.000 (0.999–1.001)	0.855		
Bilirubin	1.015 (0.968–1.064)	0.541		
PLT	1.000 (0.999–1.001)	0.889		
Creatinine	0.945 (0.872–1.024)	0.168		
BUN	0.997 (0.993–1.001)	0.174		
Hemoglobin	1.066 (1.003–1.133)	0.041		0.147
HR	1.000 (0.995–1.006)	0.933		
CHF	1.480 (1.170–1.872)	0.001		0.084
Systolic HF	3.909 (2.847–5.367)	< 0.001	5.455 (3.919–7.592)	< 0.001
Diastolic HF	2.203 (1.683–2.882)	< 0.001	3.279 (2.471–4.352)	< 0.001

OR: odds ratio; CI: Confidence interval; NE, norepinephrine; VAS, vasopressin; SOFA, sequential organ failure assessment; APS III, Acute Physiology Score III; GCS: Glasgow coma scale; PLT: Platelet; BUN, blood urea nitrogen; HR: heart rate; CHF: congestive heart failure.

Variables with *P* < 0.1 at univariable analysis were included in a multivariable logistic regression model with a stepwise selection method.

**Table 9:** Association with new-onset malignment arrhythmias among the three groups.

Parameter	Unadjusted (n = 1357)	*P*	Adjusted(n = 1357)	*P*
	OR (95% CI)		OR (95% CI)	
Gender (male)	1.491 (0.927–2.396)	0.099		0.138
Age	0.965(0.935–0.997)	0.031		0.082
Weight	1.010 (1.000–1.020)	0.059		0.065
Race(white)	1.159 (0.665–2.018)	0.603		
Group (NE reference)				
NE + VAS	3.219 (1.891–5.481)	< 0.001	3.384 (1.972–5.807)	< 0.001
Dopamine	1.208 (0.649–2.247)	0.551	1.023 0.547–1.914)	0.943
SOFA	0.957 (0.887–1.033)	0.262		
APS III	1.005 (0.995–1.016)	0.311		
GCS	0.947 (0.896–1.002)	0.057		0.448
Lactate	1.025 (0.911–1.154)	0.681		
PO_2_/FiO_2_	1.001 (0.999–1.002)	0.421		
Bilirubin	1.043 (0.966–1.125)	0.280		
PLT	1.000 (0.998–1.002)	0.874		
Creatinine	0.991 (0.835–1.176)	0.915		
BUN	1.005 (0.996–1.013)	0.280		
Hemoglobin	1.137 (1.003–1.290)	0.046		0.056
HR	1.008 (0.997–1.020)	0.163		
CHF	1.125 (0.683–1.855)	0.643		
Systolic HF	4.056 (1.622–10.145)	0.003	5.561 (2.193–14.103)	< 0.001
Diastolic HF	1.976 (1.029–3.792)	0.041	2.809 (1.444–5.465)	

OR: odds ratio; CI: Confidence interval; NE, norepinephrine; VAS, vasopressin; SOFA, sequential organ failure assessment; APS III, Acute Physiology Score III; GCS: Glasgow coma scale; PLT: Platelet; BUN, blood urea nitrogen; HR: heart rate; CHF: congestive heart failure.

Variables with *P* < 0.1 at univariable analysis were included in a multivariable logistic regression model with a stepwise selection method.

Dopamine alone had higher new-onset arrhythmias (OR = 1.665, *P* < 0.001); norepinephrine combined with vasopressin group had higher malignant arrhythmias (OR = 2.829, *P* < 0.001).

## Discussion

In our study, we found norepinephrine combined with vasopressin worsened outcomes of elderly sepsis patients with heart failure, which suggested this combination was an independent risk factor for 5-year survival. Dopamine alone reduced the Hos Los and ICU Los but had a higher risk of new-onset arrhythmias.

Population ageing is the most important medical and social demographic challenge worldwide^14^. In combination with age-related changes in the human immune system, these immunologic changes may make the elderly particularly susceptible to sepsis^15,16^. Autopsy records for elderly people over 80 in China also confirm that infection-related diseases are the second leading cause of death accounting for 26.6% of all deaths^17^. The high global mortality of sepsis^18^ is also associated with the failure to keep the hemodynamic status. The choice of vasoactive drugs is more complex and challenging, especially in sepsis patients with heart failure.

Dopamine and epinephrine are catecholamines. An early review^19^ showed that norepinephrine had an advantage over dopamine in all-cause mortality and the development of arrhythmias in septic shock. SC guidelines also recommend norepinephrine in septic shock^20^. As the first-line treatment in cardiogenic shock, norepinephrine has replaced epinephrine^21^. However, the role of which vasoactive drugs in patients with septic shock with heart failure is still controversial^22^, especially in the elderly. Vasopressin, which is synthesized by the hypothalamic paraventricular and supraoptic nucleus^23^, is recommended as second-line therapy for adults suffering from septic shock with inadequate mean artery pressure levels^24^. However, animal experiments have shown that vasopressin may decrease coronary blood flow^25^. Therefore, we would like to know if norepinephrine combined with vasopressin is appropriate for elderly sepsis patients with heart failure. We found that NE combined with vasopressin may be harmful (28-d, 90-d mortality, and other outcomes) to this study population and has the higher mortality in five-year survival analysis among three groups (*P* < 0.001). Long-term survival is independently influenced by this combination, which is not consistent with the findings of VASST in 2008^26^. However, the VASST study population did not include patients with NYHA III and IV, and patients were not grouped by age. More interestingly, in 2018, the same VASST Group found that 28-day mortality was significantly higher in NE + vasopressin group than in NE alone(60.8% vs. 46.2%, *P* = 0.009) in a retrospective study^27^. Although this retrospective analysis also did not group age and cardiac function, it has partially supported our opinion.

Second, dopamine alone shortened ICU-LOS and Hos-LOS compared with the other two groups, which sounds good for this population. After regression analysis, it was found that dopamine remained an independent risk factor for new-onset arrhythmias, which is consistent with SOAP II^22^. Meanwhile, NE + vassoprssin was the independent risk factor for new-onset malignant arrhythmias in this study population. We need to consider avoiding this combination in elderly sepsis patients with HF.

This study has the following limitations, first, we conducted a PSM analysis to minimize selection bias in a retrospective study, but the risk of residual unmeasured confounding remains possible. Therefore, the results should be considered in the target population. In addition, the limitations of this study include the lack of each patient's cardiac function and cardiorespiratory endurance before admission. Changes in blood composition may be caused by both pathogens and antibiotics. And volemic status of patients were unknown although we attempted to use CVP and CO reflect. We acknowledge that one of the limitations of our study is that data might be missing from the medical charts. Last, but not least, it was a retrospective single-center study, further multi-center prospective studies are necessary to corroborate our findings.

## Conclusions

Taken together, norepinephrine in combination with vasopressin decreased survival and increased the incidence of malignant arrhythmias in elderly sepsis patients with pre-existing heart failure. Dopamine alone reduces ICU and hospital length of stay but increases the new-onset arrhythmias.

## Data Availability

MIMIC is a public, open database for everyone. So Data for this study were obtained from the public database Medical Information Mart for Intensive Care (MIMIC III) (https://mimic.mit.edu).
